# General Decrease of Taste Sensitivity Is Related to Increase of BMI: A Simple Method to Monitor Eating Behavior

**DOI:** 10.1155/2019/2978026

**Published:** 2019-04-08

**Authors:** Arianna Vignini, Francesca Borroni, Jacopo Sabbatinelli, Sofia Pugnaloni, Sonila Alia, Marina Taus, Luigi Ferrante, Laura Mazzanti, Mara Fabri

**Affiliations:** ^1^Department of Clinical Sciences, Università Politecnica delle Marche, Via Tronto 10, 60126 Ancona, Italy; ^2^Dietology and Clinical Nutrition, Azienda Ospedaliero Universitaria Ospedali Riuniti di Ancona Umberto I Lancisi Salesi, Via Conca 71, 60126 Ancona, Italy; ^3^Center of Epidemiology, Biostatistics, and Medical Information Technology, Università Politecnica delle Marche, Via Tronto 10, 60126 Ancona, Italy; ^4^Department of Experimental and Clinical Medicine, Università Politecnica delle Marche, Via Tronto 10, 60126 Ancona, Italy

## Abstract

**Background and Objectives:**

The present study was conducted to evaluate the relationship between taste identification ability and body mass index (BMI) by studying the response to the administration of different taste stimuli to both sides of the tongue in three different groups of subjects.

**Subjects and Methods:**

Thirty healthy normal-weight volunteers, 19 healthy overweight subjects, and 22 obese subjects were enrolled. For each subject, the lateralization Oldfield score, body weight, height, and blood pressure were determined. The taste test is based on filter paper strips soaked with 4 taste stimuli presented at different concentrations to evoke 4 basic taste qualities (salty, sour, sweet, and bitter); pure rapeseed oil and water were also administered to evoke fat and neutral taste qualities. The stimuli were applied to each side of the protruded tongue. Subjects were asked to identify the taste from a list of eight descriptions according to a multiple choice paradigm.

**Results:**

The results showed a general lowering of taste sensitivity with the increase of BMI, except for the taste of fat with rapeseed oil as the stimulus. Other variables affecting taste sensitivity are age (negative association), gender (women generally show higher sensitivity), and taste stimuli concentration (positive association).

**Conclusions:**

Our findings could provide important insights into how new therapies could be designed for weight loss and long-term weight maintenance and how diets could be planned combining the correct caloric and nutritional supply with individual taste preferences.

## 1. Introduction

Obesity (OB), defined as a clinical condition characterized by an increased Body Mass Index (BMI), is a global epidemic in both children and adults. According to the World Health Organization, about 1.9 billion people in the world are overweight and at least 650 million of them are obese [1]. OB has been described as a worldwide pandemic, and the prevalence of overweight and obesity increased by 28% in adults and 47% in children between 1980 and 2013 [2]. Overweight and obesity are important public health problems because of their high prevalence in the population and their link with serious health morbidities such as hypertension, type 2 diabetes, hypercholesterolemia, cardiovascular disease, and certain types of cancers [3].

OB is fueled by individual factors, nutrition transition, and increasingly sedentary lifestyles that lead to an excessive caloric intake [4]. Among individual factors, taste identification plays an important role in food preferences, choices, and thus, consumption [5].

The taste system in humans has the peculiarity of enabling the evaluation of food for nutrients and toxicity and helping us to decide what to ingest, as well as to prepare the digestive tract for processing the nutrients introduced [6]. Humans, and possibly many other omnivores, recognize five main tastes: sweet, salty, sour, bitter, and umami. More recently, increasing evidence from human and animal studies indicate the existence of a taste modality responsive to fat, via its breakdown product fatty acids [7], and water, via aquaporines (AQPs); in particular, AQP5 has been identified in both animal and invertebrate studies [8, 9].

Although many factors contribute and affect the choice of food, such as the olfactory system, everyday life, and working habits, as well as physiological and psychological status, the present paper is focused on the analysis of the relationship between sensitivity to different tastants and BMI.

It has been suggested that altered taste perception could affect nutritional behavior and consequently body weight (or BMI). For example, with regard to sweet taste, results are contradictory; in some cases, higher glucose sensitivity has been linked to the inclination of developing obesity and diabetes [10, 11]. However, other studies have shown that there are no differences in sweet sensitivity between subjects with obesity and normal-weight individuals [12]. Concerning bitter and sour tastes, a reduction in taste sensitivity has been reported in subjects with BMI > 28 [13]. Not many works to date have studied the sensitivity for salty taste in obesity. Keskitalo et al. correlated BMI to the reported liking for salty fatty food [14], and there is a general preference by obese adults for salty food [15], thus suggesting that an alteration in salt taste sensitivity might affect eating behavior. Investigations have been conducted also on young subjects, pointing out taste sensitivity alterations in obese children and in overweight college freshmen [16, 17].

Hypothesizing that obesity negatively affects taste identification capabilities, our investigation aimed at studying the response to the administration of different tastants (citric acid, sucrose, quinine hydrochloride, sodium chloride, rapeseed oil, and deionized water) in different groups of subjects (normal weight, overweight, and obese). Moreover, we also intended to determine whether (i) increased BMI could be related to an altered taste sensitivity; (ii) such alteration affects one or more tastes (salty, sour, sweet, bitter, fat, and water); and (iii) altered taste sensitivity could lead to increased energy intake resulting in the development of obesity.

## 2. Patients and Methods

### 2.1. Patients

Thirty healthy normal-weight volunteers (18 females and 12 males, age 32.9 ± 10.0 years, BMI 21.6 ± 1.7 kg/m^2^), 19 healthy overweight subjects (11 females and 8 males, age 55.6 ± 13.6 years, BMI 27.9 ± 1.4 kg/m^2^), and 22 subjects with obesity (18 females and 4 males, age 49.0 ± 10.0 years, BMI 36.9 ± 5.7 kg/m^2^) were recruited. Obese, overweight, and control subjects' BMI were significantly different from each other. The sample size was calculated based on an expected 0.15 difference in the mean proportion of correct answers between normal-weight and overweight subjects, decreasing from a hypothesized 0.80 to 0.65, with an estimated standard deviation of 0.15 in both groups. Assuming a two-sided statistical significance of 0.05 and a power of 0.80, 16 participants per group would be needed. A sample size of at least 20 subjects per group was deemed as appropriate.

All enrolled subjects were Caucasian and were nonsmoking. Overweight and obese subjects were recruited between January and June 2016 from the Dietology and Clinical Nutrition Department at “Ospedali Riuniti” Academic Hospital, Ancona, Italy. Healthy volunteers were selected in the same period among hospital healthcare professionals and their relatives. Participants were asked to avoid eating and drinking anything except water and not to brush their teeth for one hour prior to testing. To assess the handedness of each subject, the lateralization score was determined using a 10-item inventory as described by Oldfield [18]. Body weight, height, and blood pressure were also measured at enrollment.

### 2.2. Taste Test

The taste test, performed at the Dietology and Clinical Nutrition Department, is based on filter paper strips [19] soaked with four substances (sodium chloride, citric acid, sucrose, and quinine hydrochloride) to evoke the 4 basic taste qualities (salty, sour, sweet, and bitter), each of which were presented at 4 different concentrations; in addition, pure rapeseed oil and water were administered to evoke fat taste and neutral taste, respectively. Umami was not included in the present test because the concept of this type of taste is difficult to explain and understand in Western countries. The concentrations used are shown in Table 1. Distilled water was used as a solvent, and taste solutions were freshly prepared on the morning of the testing session. The stimuli were applied to the left and right side of the protruded tongue, just posterior of the anterior third, with filter paper strips soaked in the different solutions. Before each filter paper strip application, participants were asked to wash their mouth with water. The taste presentations were randomized, and the stimulated side of the mouth was alternated, with a single trial for each combination of type of stimulus, concentration, and side of stimulation. Patients were asked to identify the taste from a list of eight descriptions, i.e., “sweet, sour, salty, bitter, oil, water, nothing, I don't know,” according to a multiple choice question.

The current study was performed in adherence to the guidelines of the Declaration of Helsinki as revised in 2001, after the protocol was approved by the Review Board of Università Politecnica delle Marche. Written informed consent was obtained from all subjects enrolled in the study prior to the anthropometric parameter measurement and execution of the taste test.

### 2.3. Statistical Analysis

Statistical analysis was conducted using IBM SPSS Statistics ver. 23.0 (IBM Co., Armonk, NY, USA). The data used in this study were answers repeatedly collected from the same subject to the various types of stimuli. Results are expressed as means ± SD. Differences were considered significant at *p* < 0.05. The Wilcoxon test was used to determine the influence of BMI on taste recognition. The *χ*^2^ test was used to assess the overall differences in categorical variables (gender and side of the tongue where stimuli were applied); the Cochran-Armitage test for trend was used to analyze the binomial outcomes across ordinal explanatory variables (BMI classes and stimuli concentrations). The multiple Pearson linear regressions were product-moment correlation coefficients computed to assess the relationships between BMI, age, and taste identification. Multivariate logistic regression was used to adjust for confounding factors identified through the results of univariate and stratified analyses.

The overall relationship between taste sensitivity and BMI and age and type of stimulation was analyzed using generalized estimating equations (GEE). In GEE, between-subject and within-subject correlations are taken into account resulting in a single regression coefficient. The repeated measurements included individuals as subject variables and type of stimulation, substance concentrations, and side of stimulation as intrasubject variables. Answers were used as dependent binary variables, assigning a value = 0 to incorrect answers and a value = 1 to correct answers. Gender, age, BMI, and characteristics of stimulation were used as independent variables. For a better presentation of the results, stimuli concentrations were log-transformed, while age and BMI were computed as a 5-unit increase. Adjusted odds ratios and standard deviations were determined with 95% confidence intervals.

## 3. Results

**Figure 1:**
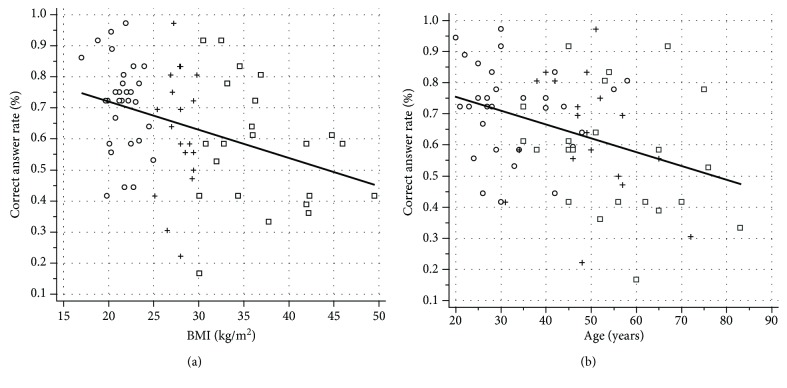
(a) Linear regression plot of BMI (kg/m^2^) and taste sensitivity, expressed as correct answer rate (*r* = −0.36; *p* = 0.002). (b) Linear regression plot of age and taste sensitivity, expressed as correct answer rate (*r* = −0.36; *p* = 0.002). Circles, control subjects; crosses, overweight subjects; squares, obese subjects.

Linear regression analysis demonstrated a general decrease of taste sensitivity with increasing BMI, shown by a reduction of the proportion of correct answers with increasing BMI (F(1, 69) = 10.441; *r* = −0.36; *p* = 0.002) (Figure 1(a)). In addition, overall gustatory sensitivity is modified in relationship to age: a negative correlation was found between the ability to correctly recognize the various tastes and age (F(1, 69) = 10.378; *r* = −0.36; *p* = 0.002) (Figure 1(b)).

### 3.1. Univariate Analysis

The results of univariate analysis showed an association between the considered variables and the response for all administered taste stimuli except for fat.

**Figure 2:**
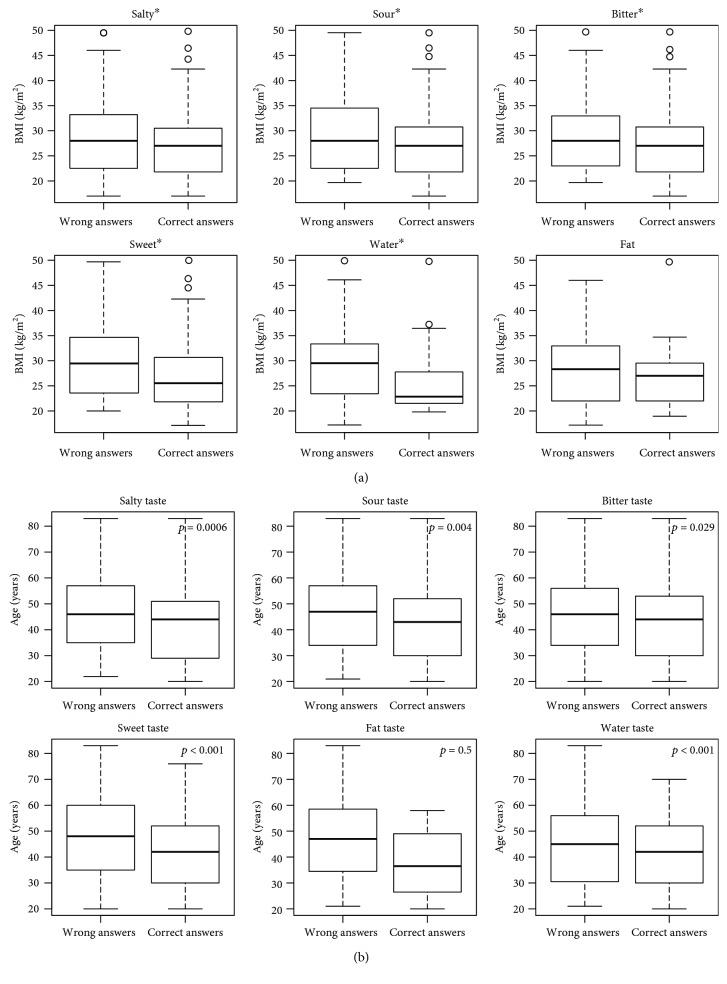
(a) Mean BMI of subjects, according to correct/wrong answers, for each type of taste stimulation. Data are expressed as mean ± SD. Asterisks (^∗^) above each graph indicate significant differences (*p* < 0.05) between groups by Wilcoxon's Signed-Ranks tests with Bonferroni correction for multiple tests. (b) Mean age of subjects, according to correct/wrong answers, for each type of taste stimulation. Data are expressed as mean ± SD. Differences (*p* < 0.05) between groups analyzed by Wilcoxon's Signed-Ranks tests with Bonferroni correction for multiple tests are highlighted in every graph.

In particular, the multiple Wilcoxon signed-rank tests, conducted for each type of taste stimulation, showed that subjects who gave a higher proportion of correct answers were characterized by significantly lower BMI (Figure 2(a)) and age (Figure 2(b)), except for fat taste, where no difference was shown.

A decreasing trend of correct responses with increasing BMI classes from normal weight to overweight was observed for sour (the Cochran-Armitage trend test, *Z* = 1.8, *p* = 0.001), bitter (*p* < 0.001), sweet (*p* < 0.001), and water (*p* < 0.01) stimuli.

Significant gender-related differences in bitter and water perception were evidenced by multiple *χ*^2^ tests conducted for each type of stimulation, and they showed a higher correct answer rate in women for bitter taste and in men for water taste. Although not significant, univariate analysis indicated that the identification of sweet taste was greater in females (Table 2).

In addition, a positive correlation between stimuli concentration and proportion of correct answers was observed for sour, bitter, and sweet stimuli (Table 2), whereas a correlation was not found regarding the side of the tongue stimulated (data not shown).

On the contrary, regarding fat taste sensitivity, the analyzed variables (BMI, age, gender, and side of the tongue) were not associated with the correct answer rate.

### 3.2. Multivariate Analysis

For each type of taste stimulation, significant variables obtained by univariate analysis were included in the multiple regression model (Table 3). Among the variables, BMI displayed a significant correlation with the correct answer rate for sour, bitter, and sweet tastes, confirming the results of the univariate analysis. Concentration, considered as -log_10_, had a significant effect on sour, bitter, and sweet taste identification. The effect of age, computed as a 5-unit increase, was significant for all types of stimulation except for bitter taste, while gender maintained significance only for bitter and water sensitivity, as indicated by the univariate analysis (see Section 3.1).

### 3.3. Generalized Estimating Equation Model

Table 4 shows the results of GEE. The results confirm that overall taste recognition capability is negatively affected by BMI and age and show a higher taste identification rate in females. Specifically, every 5-unit increase in BMI decreases the odds of correctly identifying the correct stimulus by 15%, while every 5-year increase in age decreases the odds of success by 7%. Furthermore, the rate of correct answers decreases with lower stimuli concentration.

## 4. Discussion

Obesity is the second leading cause of preventable death worldwide, and its incidence has nearly doubled since 1980. The underlying cause of obesity is a higher energy intake compared to energy expenditure, most likely due to an increased availability of palatable food. The aim of the present study was to determine the relationship, if any, between the perception of the four basic taste stimuli, water, and fat taste and BMI. The relationship was studied by analyzing the influence of the covariates age, gender, concentration of taste stimuli, and side of stimulation of the tongue on the proportion of correct answers to the administration of different taste stimuli.

The results showed a general decrease in taste sensitivity corresponding to an increase of BMI, except for fat taste. Other variables affecting taste sensitivity are age (negative association), gender (women generally show higher sensitivity), and taste stimuli concentration (positive association).

The results also showed that regarding the taste of fat, it is not possible to find any association between the considered variables and the response. Most of the participants in all groups gave no answer when stimulated with rapeseed oil; this could be related to the relatively weaker flavor and texture of this oil in comparison with more widespread vegetable oils, such as olive oil. In addition, the side of the tongue that was stimulated showed no differences in correct responses to all six taste stimuli and for all three subject populations.

The study of taste physiology and its relation to human health is receiving growing attention. It has been demonstrated that taste sensitivity is an important tool in regulating nutrient ingestion, in controlling the digestive process, and in releasing neuroendocrine hormones of hunger and satiety. Many studies have focused on changes in taste sensitivity in both physiological and pathological conditions [20–23]. Our results are in accordance with studies showing that the taste identification capacity in humans decreases with age [24–26]. Recent evidence from a large cohort of subjects demonstrated an increase in sour-bitter and bitter-sour confusion [27]. Consistently with our findings, the proportion of subjects exhibiting such confusion significantly increases with age, along with an overall impairment of taste sensitivity. However, since odor plays an important role in taste recognition, especially regarding the taste of fat [28], we need to clarify that our test was conducted with an unobstructed nasal airflow.

A previous paper by Bartoshuk et al. [29] showed that the perception of sweet and fat tastes increases as BMI increases, while Stewart et al. [30] demonstrated an impaired ability to perceive low concentrations of fatty acids with increasing BMI. Our results are inconsistent from those reported in the aforementioned papers, demonstrating a reduced sensitivity for sweet taste only (and not for fat) in overweight subjects and in subjects with obesity. Individuals who are less sensitive to sweetness could be at risk of long-term health outcomes, such as obesity and diabetes, as they will need to introduce more sugar to have the same taste sensation compared to those who are more sensitive. Concerning fat sensitivity, an increase in fat consumption and fatty food preferences may lead to the development of obesity and atherosclerosis [31]. Bitter taste perception, especially in relation to 6-n-propylthiouracil (PROP) taster status, and sour taste have also been studied in relation to BMI; both bitter and sour thresholds have been reported to be raised in subjects with obesity [13, 32]. Such results are fully in accordance with ours, showing an increased proportion of incorrect answers corresponding to the increase of BMI and a significant association of correct answers with the concentration of sour and bitter tastes.

To date, very few articles have studied savory tastes in obesity; data from those studies suggest that overweight women like salty food more than normal-weight women [33, 34]. All these studies suggest that increased BMI is associated with an alteration of taste perception.

The present data also confirm that taste recognition decreases with increasing age, in line with data present in the literature [35]. The physiological changes that occur in the taste buds are one of the main determinants of decreased taste sensitivity with increasing age [36]. Feng et al. showed that the overall number of taste buds and the number of taste cells per taste bud decrease with age, especially in men aged between 74 and 85 years [37].

Gender influence on taste recognition was also investigated in the present study: it was shown that men were better able to recognize water while women were better in recognizing bitter taste.

Taken together, all this evidence confirms that the relationship between taste sensitivity and BMI is complex. According to our results, a generalized reduction of taste identification ability was found with increasing BMI. This change may affect eating behavior and contribute to increasing calorie intake and preference for savory-salty-fatty food, which in turn may lead to overweight and obesity. However, many parameters may affect sensory processing. After ingestion, taste receptors transmit sensory signals to the brain, which segregates, evaluates, and distinguishes the stimuli, leading to the experience known as “flavor” [38]. The anatomy and physiology of taste buds, the hormonal modulation of taste function, the importance of genetic chemosensory variation, and the influence of gustatory functioning on macronutrient selection and eating behavior should be investigated in each single person, since individual genotypic variation results in specific phenotypes of food preference and nutrient intake. Analyzing the role of taste in food selection and ingestion behavior is important to expanding our understanding of the factors involved in body weight maintenance and the risk of chronic diseases including obesity, atherosclerosis, cancer, diabetes, liver disease, and hypertension.

An alternative but not contrasting hypothesis is that the general decrease of taste sensitivity, especially sweet taste, is not the cause of a BMI increase, but rather the result of a malfunctioning energy monitoring system also at brain (hypothalamic) level. This would result in an alteration of the balance between energy store and utilization. In this view, the reduced sensitivity to sweet taste could be a sign of a central homeostatic mechanism alteration, the causes of which require further investigation.

## 5. Conclusion

In conclusion, the finding of reduced taste sensitivity in obese subjects provides new evidence that could be useful for developing new strategies for weight loss and long-term weight maintenance, including dietary approaches combining correct caloric and nutritional supply with individual taste preferences. However, further investigation is certainly needed to gain additional knowledge in the field of food preferences, choice, and intake.

## Figures and Tables

**Table 1:** Characteristics of taste stimuli.

Stimulus	Substance	Concentration (g/mL)
Sweet	Sucrose	(i) 0.05 (ii) 0.1 (iii) 0.2 (iv) 0.4

Salty	Sodium chloride	(i) 0.016 (ii) 0.04 (iii) 0.1 (iv) 0.25

Bitter	Quinine hydrochloride	(i) 0.0004 (ii) 0.0009 (iii) 0.0024 (iv) 0.006

Sour	Citric acid	(i) 0.05 (ii) 0.09 (iii) 0.165 (iv) 0.3

Fat	Rapeseed oil	Pure

Neutral	Deionized water	Pure

**Table 2:** Univariate analysis.

Variable	Gender				Concentration		
Type of stimulation	Male (% correct answers)	Female (% correct answers)	*χ* ^2^	*p*	df	*χ* ^2^	*p*
Salty	62.0	65.2	0.980	0.322	3	4.722	0.193
Sour	73.9	75.4	0.112	0.738	3	25.555	<0.001
Bitter	56.0	71.5	16.914	<0.001	3	38.929	<0.001
Sweet	67.9	81.6	3.770	0.052	3	25.287	<0.001
Fat	23.9	12.5	0.524	0.469	—	—	—
Water	52.2	32.8	8.210	0.004	—	—	—

Legend: df, degrees of freedom.

**Table 3:** Multivariate analysis.

Type of stimulation	Parameter	*B*	SE	*p*	OR (CI 95%)
Salty	Age (5-unit increase)	-0.090	0.035	0.009	0.91 (0.85-0.98)
	BMI	-0.009	0.014	0.505	0.99 (0.96-1.02)

Sour	Age (5-unit increase)	-0.052	0.038	0.017	0.95 (0.88-1.02)
	BMI	-0.033	0.015	0.028	0.97 (0.94-0.99)
	Log_10_ concentration	-1.428	0.348	<0.001	0.24 (0.12-0.48)

Bitter	Age (5-unit increase)	-0.016	0.038	0.657	0.98 (0.92-1.06)
	BMI	-0.058	0.015	<0.001	0.94 (0.92-0.97)
	Log_10_ concentration	-1.372	0.227	<0.001	0.25 (0.16-0.40)
	Gender (female)	1.012	0.204	<0.001	2.75 (1.84-4.10)

Sweet	Age (5-unit increase)	-0.101	0.037	0.006	0.90 (0.84-0.97)
	BMI	-0.031	0.015	0.034	0.97 (0.94-0.99)
	Log_10_ concentration	-1.031	0.289	<0.001	0.36 (0.20-0.63)

Water	Age (5-unit increase)	-0.226	0.085	0.008	0.80 (0.67-0.94)
	BMI	-0.027	0.033	0.413	0.97 (0.91-1.04)
	Gender (female)	-1.190	0.414	0.008	0.34 (0.15-0.76)

Legend: SE, standard error; OR, odds ratio.

**Table 4:** Generalized estimated equations model.

Parameter	*B*	SE	*p*	OR (CI 95%)
Intercept	0.654	0.258	—	—
*Gender*				
Female	0.374	0.095	<0.001	1.45 (1.21-1.75)
Male	0			1
*Type of stimulation*				
Salty	2.307	0.255	<0.001	10.05 (6.09-16.57)
Sour	2.562	0.239	<0.001	12.96 (8.12-20.69)
Bitter	3.917	0.409	<0.001	50.25 (22.54-112.03)
Sweet	2.351	0.234	<0.001	10.50 (6.64-16.61)
Fat	-0.723	0.280	<0.001	0.49 (0.28-0.84)
Water	0			1
*Side of stimulation*				
Right	0.028	0.089	0.756	1.03 (0.86-1.22)
Left	0			1
*Log_10_ concentration*	-0.895	0.123	<0.001	0.41 (0.32-0.52)
*Age (5-year increase)*	-0.071	0.017	<0.001	0.93 (0.90-0.96)
*BMI (5-unit increase)*	-0.164	0.035	<0.001	0.85 (0.79-0.91)

Legend: SE, standard error; OR, odds ratio.

## Data Availability

No data were used to support this study.

## References

[1] WHO Obesity and overweight 2017. https://www.who.int/mediacentre/factsheets/fs311/en/.

[2] Smith K. B., Smith M. S. (2016). Obesity statistics. *Primary Care: Clinics in Office Practice*.

[3] D’Archivio M. (2004). *Nutritional and Genetic Factors Involved in the Obesity and in Its Clinical Complications*.

[4] French S. A., Story M., Jeffery R. W. (2001). Environmental influences on eating and physical activity. *Annual Review of Public Health*.

[5] Sørensen L. B., Møller P., Flint A., Martens M., Raben A. (2003). Effect of sensory perception of foods on appetite and food intake: a review of studies on humans. *International Journal of Obesity*.

[6] Breslin P. A. S. (2013). An evolutionary perspective on food and human taste. *Current Biology*.

[7] Liu D., Archer N., Duesing K., Hannan G., Keast R. (2016). Mechanism of fat taste perception: association with diet and obesity. *Progress in Lipid Research*.

[8] Watson K. J., Kim I., Baquero A. F., Burks C. A., Liu L., Gilbertson T. A. (2007). Expression of aquaporin water channels in rat taste buds. *Chemical Senses*.

[9] Solari P., Masala C., Falchi A. M., Sollai G., Liscia A. (2010). The sense of water in the blowfly *Protophormia terraenovae*. *Journal of Insect Physiology*.

[10] Perros P., MacFarlane T. W., Counsell C., Frier B. M. (1996). Altered taste sensation in newly-diagnosed NIDDM. *Diabetes Care*.

[11] Schiffman S. S., Graham B. G., Sattely-Miller E. A., Peterson-Dancy M. (2000). Elevated and sustained desire for sweet taste in African-Americans: a potential factor in the development of obesity. *Nutrition*.

[12] Anderson G. H. (1995). Sugars, sweetness, and food intake. *The American Journal of Clinical Nutrition*.

[13] Simchen U., Koebnick C., Hoyer S., Issanchou S., Zunft H. J. F. (2006). Odour and taste sensitivity is associated with body weight and extent of misreporting of body weight. *European Journal of Clinical Nutrition*.

[14] Keskitalo K., Tuorila H., Spector T. D. (2008). The Three-Factor Eating Questionnaire, body mass index, and responses to sweet and salty fatty foods: a twin study of genetic and environmental associations. *The American Journal of Clinical Nutrition*.

[15] Cox D. N., Perry L., Moore P. B., Vallis L., Mela D. J. (1999). Sensory and hedonic associations with macronutrient and energy intakes of lean and obese consumers. *International Journal of Obesity*.

[16] Noel C. A., Cassano P. A., Dando R. (2017). College-aged males experience attenuated sweet and salty taste with modest weight gain. *The Journal of Nutrition*.

[17] Overberg J., Hummel T., Krude H., Wiegand S. (2012). Differences in taste sensitivity between obese and non-obese children and adolescents. *Archives of Disease in Childhood*.

[18] Oldfield R. C. (1971). The assessment and analysis of handedness: the Edinburgh inventory. *Neuropsychologia*.

[19] Landis B. N., Welge-Luessen A., Brämerson A. (2009). “Taste strips”—a rapid, lateralized, gustatory bedside identification test based on impregnated filter papers. *Journal of Neurology*.

[20] Wise P. M., Breslin P. A. S. (2013). Individual differences in sour and salt sensitivity: detection and quality recognition thresholds for citric acid and sodium chloride. *Chemical Senses*.

[21] Rabin M., Poli de Figueiredo C. E., Wagner M. B., Antonello I. C. F. (2009). Salt taste sensitivity threshold and exercise-induced hypertension. *Appetite*.

[22] Correa M., Laing D. G., Hutchinson I., Jinks A. L., Armstrong J. E., Kainer G. (2015). Reduced taste function and taste papillae density in children with chronic kidney disease. *Pediatric Nephrology*.

[23] Negri R., di Feola M., di Domenico S. (2012). Taste perception and food choices. *Journal of Pediatric Gastroenterology and Nutrition*.

[24] Stevens J. C., Cain W. S. (1993). Changes in taste and flavor in aging. *Critical Reviews in Food Science and Nutrition*.

[25] Heft M. W., Robinson M. E. (2014). Age differences in suprathreshold sensory function. *Age*.

[26] Solemdal K., Sandvik L., Willumsen T., Mowe M. (2014). Taste ability in hospitalised older people compared with healthy, age-matched controls. *Gerodontology*.

[27] Doty R. L., Chen J. H., Overend J. (2017). Taste quality confusions: influences of age, smoking, PTC taster status, and other subject characteristics. *Perception*.

[28] Ebba S., Abarintos R. A., Kim D. G. (2012). The examination of fatty acid taste with edible strips. *Physiology & Behavior*.

[29] Bartoshuk L. M., Duffy V. B., Hayes J. E., Moskowitz H. R., Snyder D. J. (2006). Psychophysics of sweet and fat perception in obesity: problems, solutions and new perspectives. *Philosophical Transactions of the Royal Society B: Biological Sciences*.

[30] Stewart J. E., Newman L. P., Keast R. S. J. (2011). Oral sensitivity to oleic acid is associated with fat intake and body mass index. *Clinical Nutrition*.

[31] Newman L., Haryono R., Keast R. (2013). Functionality of fatty acid chemoreception: a potential factor in the development of obesity?. *Nutrients*.

[32] Tepper B. J., Banni S., Melis M., Crnjar R., Tomassini Barbarossa I. (2014). Genetic sensitivity to the bitter taste of 6-*n*-propylthiouracil (PROP) and its association with physiological mechanisms controlling body mass index (BMI). *Nutrients*.

[33] Beauchamp G. K. (2009). Sensory and receptor responses to umami: an overview of pioneering work. *The American Journal of Clinical Nutrition*.

[34] Pepino M. Y., Finkbeiner S., Beauchamp G. K., Mennella J. A. (2010). Obese women have lower monosodium glutamate taste sensitivity and prefer higher concentrations than do normal-weight women. *Obesity*.

[35] Uota M., Ogawa T., Ikebe K. (2016). Factors related to taste sensitivity in elderly: cross-sectional findings from SONIC study. *Journal of Oral Rehabilitation*.

[36] Ogawa T., Annear M. J., Ikebe K., Maeda Y. (2017). Taste-related sensations in old age. *Journal of Oral Rehabilitation*.

[37] Feng P., Huang L., Wang H. (2013). Taste bud homeostasis in health, disease, and aging. *Chemical Senses*.

[38] Loper H. B., La Sala M., Dotson C., Steinle N. (2015). Taste perception, associated hormonal modulation, and nutrient intake. *Nutrition Reviews*.

